# Chronotype as a predictor of athletic performance in youth with mild intellectual disabilities

**DOI:** 10.3389/fphys.2024.1405595

**Published:** 2024-07-05

**Authors:** Ahmet Kurtoğlu, Özgür Eken, Musa Türkmen, Bekir Çar, Edi Setiawan, Baglan Yermakhanov, Madawi H. Alotaibi, Safaa M. Elkholi

**Affiliations:** ^1^ Department of Coaching Education, Faculty of Sport Science, Bandirma Onyedi Eylul University, Balikesir, Türkiye; ^2^ Department of Physical Education and Sport Teaching, Faculty of Sport Sciences, Inonu University, Malatya, Türkiye; ^3^ Department of Physical Education and Sport Teaching, Faculty of Sport Sciences, Bandirma Onyedi Eylul University, Bandirma, Türkiye; ^4^ Faculty of Teacher Training and Education, Universitas Suryakancana, Cianjur, Indonesia; ^5^ Faculty of Sports and Arts, Khoja Akhmet Yassawi International Kazakh-Turkish University, Turkestan, Kazakhstan; ^6^ Department of Rehabilitation Sciences, College of Health and Rehabilitation Sciences, Princess Nourah bint Abdulrahman University, Riyadh, Saudi Arabia

**Keywords:** athletic performance, chronotype, circadian rhythm, exercise, intellectual disability

## Abstract

**Aim:**

This study aimed to explore the influence of circadian rhythms on athletic performance in individuals with mild intellectual disabilities (ID), with a specific focus on elucidating the association between chronotype and various performance metrics.

**Methods:**

The study was a cross-sectional study consisting of 30 male participants aged between 11 and 19 years and diagnosed with mild ID. The chronotypes of all participants were assessed using the Childhood Chronotype Questionnaire (CCQ). Performance assessments were divided into three groups. Group A tests [sit and reach, medicine ball throw (MBT), plank], group B tests [handgrip strength (HGS), standing long jump (SLJ), 20-m sprint (20 ms)] and group C tests [vertical jump (VJ), hanging with bent arm (HBA), Illinois agility test) in order to ensure adequate rest periods between tests and not to affect the results. These group tests were performed 48 h apart, between 09:00–10:00 and 17:00–18:00, after a dynamic warm-up session.

**Results:**

Significant variations were observed in the sit-and-reach test (t = −4.154, d = −0.75, *p* < .001), HGS (t = −2.484, d = −0.45, *p* = .019), SLJ (t = −2.117, d = −0.38, *p* = .043), VJ (t = −5.004, d = −0.91, *p* < .001), and plank duration (t = −4.653, d = −0.84, *p* < .001). Evening performances showed improvement in MBT, HBA, 20 ms, and the Illinois agility test, although these differences were not statistically significant (*p* > .05). Notably, positive correlations were identified between participants’ chronotypes and their performance in HBA (morning/evening; r = .693, *p* = .026; r = .656, *p* = .039, respectively) and the plank (evening; r = .717, *p* = .020), with negative correlations noted in the 20 ms (morning/evening; r = −.703, *p* = .023; r = −.710, *p* = .021, respectively).

**Conclusion:**

The findings suggest that individuals with mild ID exhibit enhanced athletic performance during evening hours. These insights underscore the importance of considering chronotype in tailoring exercise interventions for this population to optimize outcomes.

## 1 Introduction

Intellectual disability (ID) is defined as a persistent loss of mental functions (Linden, 2017). This impairment of mental functions is associated with the brain’s frontal lobe and prefrontal cortex. The frontal lobe of the brain is responsible for organizing the data flow from the limbic system, sensory system, and subcortical structures, and manages tasks such as planning, coping with problems, attention, and motor functions. The prefrontal cortex, located in the anterior region of the frontal lobe, is tasked with coordinating complex information networks and regulating the connections between sensory and motor systems (Royall et al., 2002). Damage to the brain’s frontal lobe and prefrontal cortex can limit an individual’s executive functions such as motor control, learning, reasoning, directing attention, and suppressing unwanted behaviors (Hill, 2004). Given that ID restricts the functions of brain activities, we hypothesize that individuals with ID may exhibit certain variations in their biological rhythms.

Biological rhythms are the cyclical responses of living organisms to environmental stimuli (Allebrandt et al., 2014; Bayer et al., 2023). The adaptation to these stimuli and their optimal utilization aid in biochemical reactions and maintaining the body’s equilibrium. Examples of such environmental stimuli include the day-night cycle, seasonal changes, and the tidal movements influenced by the moon (Refinetti, 2008). The circadian rhythm, known as the day-night cycle, reflects the metabolic changes in organisms during a full rotation of the Earth on its axis (24 h) (Sozlu and Sanlier, 2017). This rhythm is regulated by the suprachiasmatic nucleus (SCN) located in the hypothalamus of the brain (Moore, 2007; Vitale and Weydahl, 2017a; Postolache et al., 2020). Structures such as the pineal gland, pituitary gland, olfactory bulb, ventral hypothalamus, proventricular nucleus, and arcuate nucleus are associated with the circadian cycle and exhibit increased activity in the evening (Abe et al., 2002). The circadian rhythm persists even in the absence of environmental factors or when opposite environmental stimuli are presented (Marques, 2013). Factors like light, darkness, temperature, and jet lag can disrupt the circadian rhythm, causing deviations in the 24-h cycle (Lack et al., 2009). Therefore, a thorough analysis of the effects of environmental and individual factors on the circadian rhythm is imperative. Such an analysis can provide a multifaceted understanding of the circadian variations in athletic performance.

Cyclically recurring daily changes significantly impact physical performance. Studies designed with circadian rhythms in consideration have examined variations in athletic performance across morning, noon, and evening (Kurtoglu, 2023; Nobari et al., 2023; Ouergui et al., 2023). The physiological state and performance output of individuals vary throughout the day, often showing differences from morning to evening. Numerous studies have identified enhanced performance levels in the evening (López-Samanes et al., 2017; Ouergui et al., 2023). Some research has focused on the influence of an individual’s chronotype (morning, evening, or neither) in determining their peak performance time (Montaruli et al., 2021). In their systematic review, Vitale and Weydahl reported that morning-type individuals exhibited higher performance in the morning compared to evening types. They also concluded that physiological responses to physical activity yielded inconsistent results due to heterogeneous groups and varying types of physical activities (Vitale and Weydahl, 2017a). These findings demonstrate that the circadian rhythm operates like a biological clock, with the brain as its central hub. Therefore, elucidating the relationship between the loss of brain functions in individuals with intellectual disabilities (ID) and the circadian rhythm through athletic performance indicators represents a significant contribution to the literature.

Although there are many studies on diurnal variability and circadian rhythm in healthy populations, the number of studies investigating these factors in individuals with ID is limited. At the same time, there is no research examining the diurnal variability of performance indicators in individuals with ID. It is important to analyze these variables to optimize the athletic performance of individuals with ID and to provide maximum benefit during exercise. Therefore, this study aimed to investigate the effect of diurnal variability on athletic performance indicators in individuals with ID. As a result, the hypothesis of our study was “Athletic performance parameters change from morning to evening in individuals with mild ID”.

## 2 Methods

### 2.1 Participants

This study employed quantitative and experimental methods. 30 male participants with mild ID were included using simple random sampling from a special education institution. The participants’ ages averaged 15.20 ± 2.32 years, with heights of 1.63 ± 0.09 m, weights of 66.86 ± 16.58 kg, and Body Mass Index (BMI) ranging between 24.79 ± 4.94 kg/m^2^. These participants were diagnosed with mild ID by specialist teams operating in Guidance and Research Centers, using standardized tests. The diagnosis of mild intellectual disability is determined based on IQ level, with the American Association on Intellectual and Developmental Disabilities (AAIDD) setting the IQ threshold for mild intellectual disability between 55–70 points (Smit et al., 2019). In this context, individuals with mild intellectual disabilities with IQ levels between 55 and 70 who were studying at the high school level participated in our study. Individuals who had undergone surgery, had a physical disability, or had heart disease, diabetes, or hypertension within the last year were excluded from the study. It was also confirmed that the participants did not consume alcohol or tobacco (Reilly et al., 2007). Medical histories and additional information about the participants were obtained from their families. Before the tests, individuals reported no sleep deprivation, anxiety, or acute problems. At the same time, the parents of the participants verbally reported that the participants had at least 8 hours of sleep in the evening and that they had avoided food and beverages that would affect performance at least 3 h before the tests. Participants who did not fulfill these criteria and participants who did not comply with the principal investigator’s commands were excluded from the study.

In this study, the G-Power software (version 3.1.9.7) was utilized to determine the minimum sample size of participants. A reference study by Jarraya et al. (2015), which analyzed the diurnal variation in physical performance of Tunisian children, recorded morning running performance at 22.9 ± 0.9 s and evening performance at 22.1 ± 0.9 s (Jarraya et al., 2015). Based on this, a power analysis was conducted using the *t*-test (Means: Difference between two dependent means (matched pairs), *A priori*: compute required sample size-given α, power, and effect size). The parameters were set as α = 0.05, power (1-β err prob) = 0.8, and effect size = 0.38, which determined that a minimum of 10 participants was required for an actual power of 82.7%.

Participants and their families were informed about the purpose, rationale, and expected contribution to the research. Following this, written and signed voluntary consent forms were obtained from all participants and their parents. Necessary permissions were acquired from the relevant institutions. This research was approved by the Non-Interventional Ethical Committee of Bandirma Onyedi Eylul University under decision number 2022/153. Additionally, the study was conducted by the principles outlined in the Helsinki Declaration.

### 2.2 Experimental design

In our research, to mitigate the potential influence of simultaneous testing on the findings, the nine tests conducted were divided into three groups: A, B, and C. This categorization was meticulously designed to ensure homogeneity in the energy capacity required for each test. One week before the testing, participants were briefed about the tests to ensure familiarity and to prevent any procedural issues during implementation. All tests were demonstrated by the responsible researcher using the “show and tell” technique, after which all participants were asked to replicate the tests.

**FIGURE 1:**
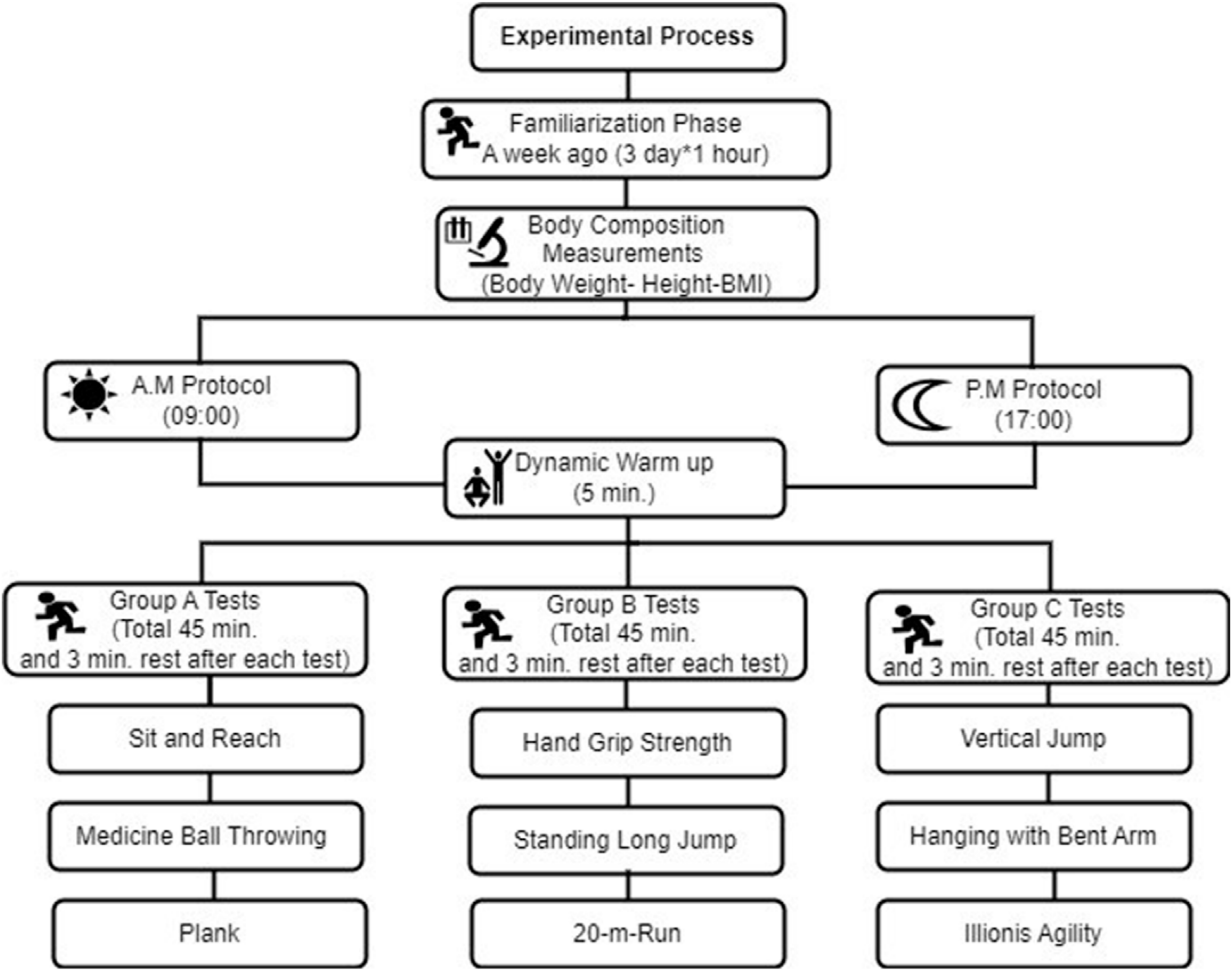
Experimental design of study.

Additionally, participants’ chronotypes were determined using a scale developed by Zavada et al. (2005). (Zavada et al., 2005). According to this, two participants were categorized as morningness (20%), seven as intermediate type (70%), and one as eveningness (10%). After a familiarization period of 3 days (1 h each day), the participants were capable of performing the tests. Tests from groups A, B, and C were administered at a 48-h interval, at 09:00 a.m. and 05:00 p.m., respectively. The total duration of each group’s tests was 45 min. A 5-min dynamic warm-up was conducted before the tests, followed by a 3-min rest period after each test. The test content included: Group A with the Sit and Reach Test, Medicine Ball Throw, and Plank; Group B with Handgrip Strength, Standing Long Jump, and 20-m Sprint; and Group C with Vertical Jump, Bent Arm Hang, and Illinois Agility Test. For test standardization, participants were numbered from 1 to 10. Care was taken to ensure that the same sequence of participants was maintained for both morning and evening tests (Figure 1). One day before the commencement of the study, participants were advised to avoid moderate and high-intensity activities, abstain from caffeine, ensure at least 7–8 h of sleep, and not eat anything within 2 h before the tests.

### 2.3 Data collection tools

Participants’ weights were measured using a Medisana ZFC5087 electronic scale with a precision of 0.1 kg/0.2 lb. Their heights were determined using a tape measure with an accuracy of 0.1 cm. During height measurement, participants were instructed to stand straight with their heads upright and legs fully extended (Tamer, 2000). Body Mass Index (BMI) values were calculated using the formula weight/height (kg/m^2^) (Nihiser et al., 2007). A 5-min dynamic warm-up session was conducted before the tests.

### 2.4 Childhood chronotype questionnaire (CCQ)

The scale used to determine the participants’ chronotype was originally developed by Zavada et al. (2005) (Zavada et al., 2005). Its validity and reliability studies in Turkey were conducted by Dursun et al. (2015) (Dursun et al., 2015). The questionnaire consists of 27 items, with questions 16–26 calculating the total score for morningness/eveningness. Scores of ≤23 indicated morningness, 24–32 intermediate type, and ≥33 eveningness. The questionnaire was completed by the participants’ families before the tests.

### 2.5 Sit and reach test

Participants were instructed to sit on the floor with their legs extended forward. Their toes were positioned upwards, and a distance of 30–40 cm was maintained between their heels. A straight strip was placed between the two feet, and another strip measuring 60–80 cm in length was positioned perpendicularly from the midpoint of the first strip. Participants were asked to reach forward as far as they could without bending their knees, with pressure applied to the knees to ensure they remained straight. The best score of two attempts was recorded in centimeters (Cuberek et al., 2013). Each attempt was completed within 20 s. For each participant, the total duration for two attempts was 40 s, resulting in an approximate total time of 7 min for all participants.

### 2.6 Handgrip strength (HGS)

The handgrip strength of participants’ right hands was measured using a Smedley Electronic Hand Dynamometer with a sensitivity of 0.1 kg. Measurements were taken with the individual’s shoulder in a normal position and the elbow at a 90° flexion (Shechtman et al., 2004). The best of three attempts, conducted at 30-s intervals, was recorded. Each attempt lasted 15 s. The total duration for each participant was approximately 1 min and 45 s, resulting in an overall time of about 18 min for all participants.

### 2.7 Standing long jump (SLJ)

This test was performed on a non-slip, firm surface. Participants stood with their feet together, toes behind the jump line. They jumped as far as possible from a bent-knee position with swinging arms. The best score of two attempts, measured with a meter and conducted at 30-s intervals, was recorded in centimeters (Ruiz et al., 2011). Each attempt lasted 15 s. The total duration for each participant was approximately 1 min and 30 s, resulting in an overall time of about 15 min for all participants.

### 2.8 Medicine ball throw (MBT)

Participants positioned themselves at the throwing line, ensuring their toes did not cross it. They threw a medicine ball as far as possible without using their lower extremities. The best score of three attempts, conducted at 30-s intervals, was recorded in centimeters (Mills et al., 2020). Each throw took about 20 s, with a 30-s rest period after each attempt. The total duration for each participant was 2 min, amounting to 20 min for all participants.

### 2.9 Bent arm hang (BAH)

This test was performed using a 2.5 cm diameter pull-up bar. Participants gripped the bar with thumbs under and other fingers over, shoulder-width apart. The test ended when the participant’s eyes dropped below the level of the bar. The best of two attempts was timed with a stopwatch (Getchell, 1979). Each attempt lasted approximately 15 s, with a total duration of 30 s for two attempts and a 30-s rest in between. The overall duration of the test was approximately 9 min.

### 2.10 Vertical jump (VJ)

The test was carried out against a flat wall. The participant’s maximum reach was measured using a tape measure. The participant then jumped from a standing position, reaching as high as possible. The highest difference between the two attempts was recorded in centimeters (Aragón, 2000). Each attempt took about 20 s, with a total duration of 40 s for two attempts and a 30-s rest in between. The total time for all participants was approximately 12 min.

### 2.11 Plank

Participants adopted a face-down position, supporting themselves on forearms and feet, with the core area not touching the ground. The head, spine, and ankles were aligned. The duration for which the position was maintained was timed (Boyer et al., 2013). The plank test took approximately 15 min in total.

### 2.12 20-m sprint (20 mS)

This test was conducted on a 20-m track. Participants started in a running position, with the body at approximately 45° flexion. They sprinted the distance at maximum speed upon a signal. The best time of two attempts, conducted with full rest in between, was recorded with a stopwatch. Each attempt took about 10 s, totaling 20 s for two attempts. Each participant’s attempts were spaced by approximately 3 min. The total duration for all participants was about 8 min.

### 2.13 Illinois agility test

Conducted on a standard 5 m by 10 m field with markers 3.3 m apart, participants started in a similar running position as the 20-m sprint. They completed the course at maximum speed after a signal. The best time of two attempts was recorded with a stopwatch (Getchell, 1979). Each attempt took about 35 s, totaling 1 min and 10 s for two attempts. Each participant’s attempts were spaced by approximately 3 min. The total duration for all participants was about 15 min.

### 2.14 Statistical analysis

Statistical analysis was performed using the SPSS 25.0 package program. Shapiro Wilk test was used to determine whether the data were normally distributed. In our study, homogeneity of variances was determined by Levene’s test. Accordingly, it was determined that the data were normally distributed. It was determined that the data were normally distributed. Therefore, a Paired Sample *t*-Test was performed to compare morning and evening performance parameters. After the chronotypes of the participants (morning and evening) were determined with the CCQ, Pearson Correlation analysis was performed to analyze the relationship between the sit-reach, HGS, SLJ, MBT, BAH, VJ, plank, 20 ms, and Illinois agility test results. GraphPad Prism 8 software was used for graphical operations. To calculate the effect size, Cohen’s d effect size (ES), which divides the difference between two averages by the standard deviation of the data, was calculated using the following criteria: 0.2 is considered as small, 0.5 as medium, and 0.8 as significant (Cohen, 1988). The significance level in the study was set at 0.05.

## 3 Results

**FIGURE 2:**
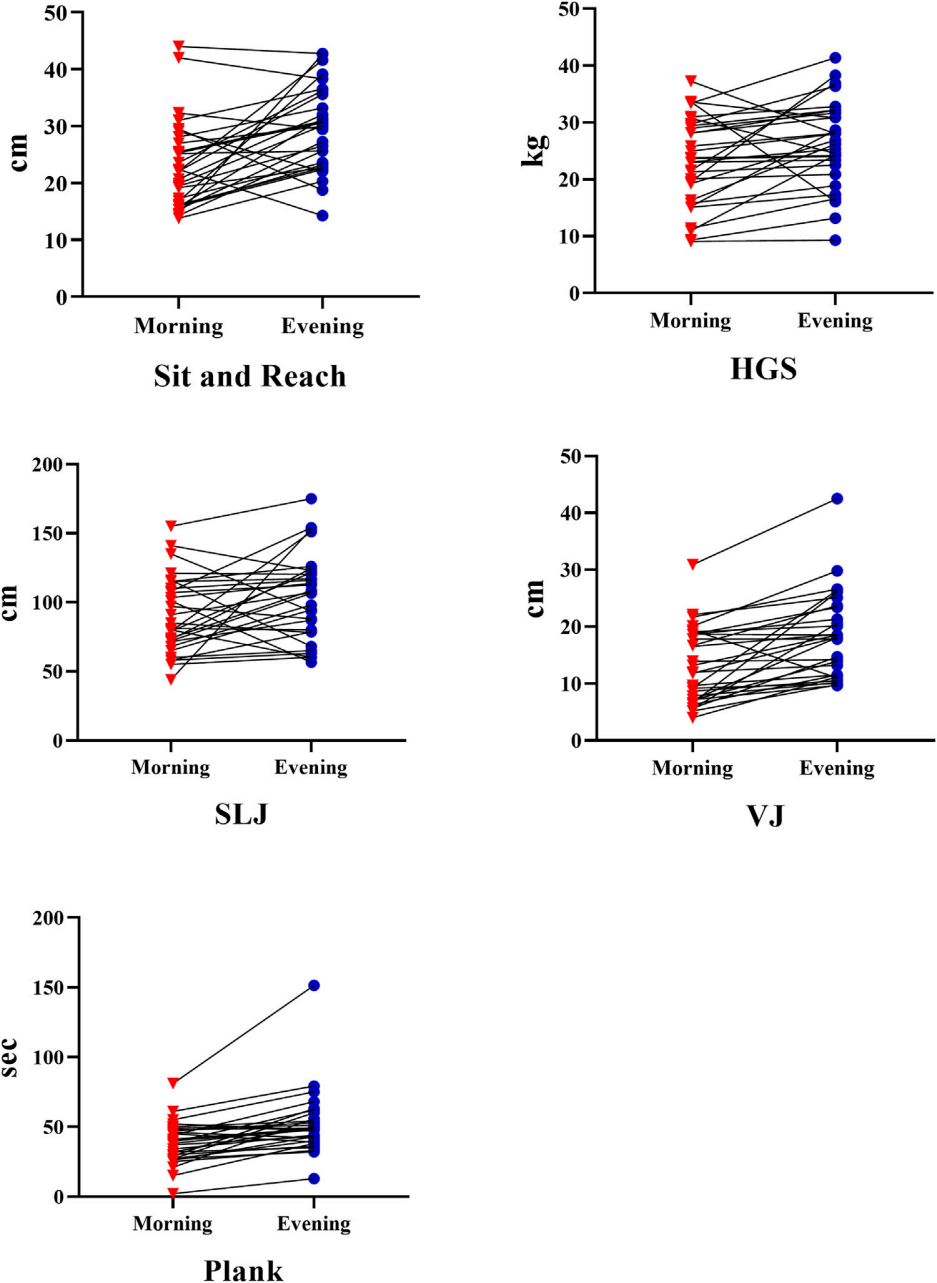
Participants performance results from morning to evening: HGS hand standing long jump; VJ; vertical jump.

Table 1 shows the performance of the participants from morning to evening. Accordingly, the participants’ sit reach test results (t = −4.154, d = −0.75, *p* < .001), HGS results (t = −2.484, d = −0.45, *p* = .019), SLJ results (t = −2.117, d = −0.38, *p* = .043), VJ results (t = −5.004, d = −0.91, *p* < .001), and plank duration results (t = −4.653, d = −0.84, *p* < .001) increased significantly from morning to evening (Figure 2). In contrast, the MBT, HBA, 20 ms, and Illinois tests showed no change from morning to evening (*p* > 0.05).

**TABLE 1:** Analysis of participants’ performance from morning to evening.

Parameters	Morning M±S.D.	Evening M±S.D.	t	Cohen’s d	p
Sit and Reach (cm)	22.99 ± 7.65	29.53 ± 7.36	−4.154	−0.75	<.001**
HGS (kg)	23.12 ± 7.80	26.33 ± 7.53	−2.484	−0.45	0.019*
SLJ (cm)	90.59 ± 27.63	103.33 ± 30.94	−2.117	−0.38	.043*
MBT (cm)	350.46 ± 86.67	373.65 ± 85.96	−1.579	−0.28	.125
HBA (sec)	8.17 ± 11.24	8.64 ± 10.69	−0.898	−0.16	.377
VJ (cm)	13.02 ± 6.64	18.22 ± 7.53	−5.004	−0.91	<.001**
Plank (sec)	38.41 ± 15.18	51.67 ± 23.09	−4.653	−0.84	<.001**
20 mS (sec)	4.83 ± 0.28	4.70 ± 0.38	1.837	0.33	.077
İllinois Test (sec)	26.83 ± 5.55	27.37 ± 3.25	−0.795	0.14	.433

HGS, hand grip strength; SLJ, standing long jump; MBT, medicine ball throwing; HBA, hanging with bent arm; VJ, vertical jump; 20 ms: 20 m Sprint; **p < .001; *p < .05.

Table 2 shows the results of Pearson Correlation analysis of the relationship between the participants’ chronotype and performance parameters. According to the results, HBA-morning (r = .693, *p* = .026), HBA-evening (r = .656, *p* = .039) and plank-evening (r = .717, *p* = .020) were positively correlated with morning-to-evening chronotype. 20mS-morning (r = −.703, *p* = .023) and 20 mS-evening (r = −.710, *p* = .021) were negatively associated with morning-to-evening chronotype. There was no significant correlation between chronotype and other parameters (*p* > .05).

**TABLE 2:** Pearson Correlation Analysis Results between chronotypes and performance parameters.

Parameters	Chronotype
HBA- morning	r = .693, *p* = .026
HBA- evening	r = .656, *p* = .039
Plank-evening	r = .717, *p* = .020
20 mS-morning	r = −.703, *p* = .023
20 mS-evening	r = −.710, *p* = .021

HBA: hanging with bent arm, 20 mS: 20 m Sprint.

## 4 Discussion

In our study, which examined the changes in some performance analysis of individuals with ID from morning to evening, it was concluded that the results of sit and reach, HGS, SLJ, VJ, and plank test increased significantly from morning to evening. It was also found that most of the participants had an intermediate chronotype. There was a positive correlation between these chronotypes and HBA, plank, and a negative correlation between 20 ms performance. The results of our study are important for analyzing the effects of brain function on circadian rhythm in individuals with ID. Therefore, our hypothesis “Athletic performance parameters differ from morning to evening in individuals with ID about changes in brain function” was confirmed. To the best of our knowledge, this is the first study to analyze the change in athletic performance parameters from morning to evening in individuals with ID.

The suprachiasmatic nucleus (SCN), located in the anterior part of the hypothalamus, is a bilateral structure and the primary determinant of circadian rhythm timing. Most afferent neuronal pathways extend to the SCN, and disruptions in the circadian rhythm are associated with sleep and mood disorders (Ma and Morrison, 2023). It is known that sleep problems occur in 13%–86% of individuals with ID (Didden et al., 2002; Bohmer et al., 2022). These wide-ranging variations in sleep disturbances are believed to cause alterations in the circadian rhythms of individuals with ID (Braam et al., 2009). In their study, Ballester-Navarro et al., 2021 attempted to identify the interaction between the circadian clock and melatonin pathway gene variants in ID individuals with sleep issues. They suggested that normal and abnormal sleep-wake rhythms might be associated with circadian clock and melatonin pathway gene variants (Ballester-Navarro et al., 2021). Furthermore, research with rats has reported that the sleep-wake cycle is controlled in the cortex, cerebellum, forebrain, and hypothalamus (Terao et al., 2003; Cirelli et al., 2004). This could be one reason for the heterogeneous results of studies examining athletic performance levels about circadian rhythms. However, our findings of increased performance parameters in the evening are consistent with studies conducted on healthy individuals, particularly reporting higher performance levels around 16:30–19:00 (Aloui et al., 2017; Cavalcante-Silva et al., 2018; di Cagno et al., 2013; Rakita et al., 2020). This suggests that the brain physiology of individuals with ID may operate similarly to that of healthy individuals. However, studies are reporting age-related variations in this difference. Surtees et al., 2018 conducted a meta-analysis on sleep duration and quality in ID and non-ID individuals. They reported that individuals with ID slept 18 min less than healthy individuals, with no evidence to suggest this difference diminishes in adulthood (Surtees et al., 2018). These results contradict studies claiming the degeneration of the SCN, the primary timer of the circadian rhythm, with aging. Hoffman’s research reported that vasopressin immunoreactive neurons in the SCN showed marked oscillation in young individuals but no significant changes in older adults. It was concluded that vasopressin synthesis displays a disrupted circadian rhythm in later life (Hofman and Swaab, 1994). Since our study sample comprised individuals aged 11–19 with mild ID, perhaps the similar results to those of healthy individuals are due to the ongoing activity of vasopressin immunoreactive neurons in the SCN.

Maaskant et al., 2013 have reported that individuals with ID experience a type of brain dysfunction, which likely affects the sleep-wake cycle in the suprachiasmatic nucleus (SCN) and consequently, the circadian rhythm. This finding appears to contradict the results of our study. A potential explanation for this discrepancy could be that the sample in Maaskant et al.’s study included individuals with conditions like autism, dementia, and Down syndrome, who face more severe cognitive challenges compared to those with mild ID. Supporting this notion, Patel et al., 2020 indicated that individuals with severe ID are characterized by genetic abnormalities, congenital metabolic errors, and brain malformations. Additionally, several studies have noted that over 75% of individuals with severe ID have specific genetic, biological, or neurological conditions (Maulik et al., 2011; Moeschler et al., 2014; Huang et al., 2016). Therefore, it is hypothesized that studies conducted on individuals with moderate to severe ID might yield different results.

Chronotype refers to an individual’s preferred sleep-wake schedule and is associated with the temporal organization of mental and physical activities, sleep and wake times, usual meal times, mood, and biological variables throughout the day (Brito et al., 2022). The frequent occurrence of sleep disorders in individuals with ID (Didden et al., 2002; Böhmer et al., 2022), suggests that these individuals may also exhibit varied chronotypes. Consequently, it is commonly understood that individuals with ID, often displaying eveningness chronotypes due to their tendency to sleep late and wake up late, may experience certain mood disorders and physical abnormalities (Türkoğlu and Çetin, 2019). Some studies have found that individuals with an eveningness chronotype exhibit higher performance levels in the evening. Research by Facer-Childs et al., 2018 indicated that individuals with an eveningness chronotype performed 8.4% worse in the morning. Another study by Anderson et al., 2018 revealed that evening-type swimmers were 6% slower in the mornings, while morning-type individuals required 5–7 times more energy to match their morning performance in the evenings. This is thought to be due to the SCN’s regulation of body temperature, hormone secretion, and sleep-wake balance by synchronizing the biological rhythm according to environmental light-wave patterns. This regulation also influences the release of hormones such as melatonin and cortisol, optimizing a healthy nervous system functioning in the organism (Rosenwasser and Turek, 2015). Despite most of our study’s sample having an intermediate chronotype, performance parameters increased in the evening hours. Our correlation analysis found positive correlations between HBA and plank performances with chronotype, and a negative correlation between 20 m performance and chronotype. This suggests that more research is needed to investigate the circadian changes in individuals with ID according to chronotype.

It is known that individuals with ID have some problems in motor skills, the neuromuscular system, and the sensory-motor system (Carmeli et al., 2008; De Giorgio et al., 2017; Borji et al., 2021). However, it is very important to understand the relationship of performance with chronotype and circadian rhythm in individuals with ID. When analyzed in this context, a systematic review examining the effect of chronotype on performance parameters in healthy individuals reported that the performance levels of morning types were higher in the morning hours, but other results regarding the effect of chronotype on physiological responses to physical activity were not always consistent. He argued that this was due to heterogeneous sample groups and different types of physical activity (Vitale and Weydahl, 2017a). In our study, sit and reach, HGS, SLJ, VJ, and plank levels were significantly higher in the evening. In our study, sit and reach, HGS, SLJ, VJ, and plank levels were significantly higher in the evening. In the meta-analysis conducted by Knaier et al., 2022 it was concluded that maximum power, HGS and jump height were higher in the evening hours. In the study conducted by Atan et al., 2017 it was concluded that the best performance levels of the participants in the 20 m shuttle run test were in the afternoon. It was reported that there was no difference between morning and evening (Atan et al., 2017). The fact that there were no measurements in the afternoon in our study confirms the similarity between morning and evening. When these results were evaluated, it was determined that different results were obtained in studies examining athletic performance levels from morning to evening, whether in athlete populations or in healthy individuals. When compared with our results, although similar results were obtained with the studies in the literature, randomized controlled studies are needed to investigate the circadian mechanism and chronotype in individuals with ID.

## 5 Study limitation

This study was conducted only on individuals with mild ID. Future studies on individuals with different ID classifications may better analyze the effects of increasing ID levels on brain function and circadian rhythm. Another important limitation of our study is the small number of samples in different chronotypes. This made it difficult to perform statistical procedures between chronotypes. Another limitation of this study is that the sample consists of only male individuals with ID. For this reason, it is thought that there is a need for research results that also include individuals with female ID. In our study, the sleep-wakefulness results of the participants were self-reported using the CCQ scale. More important results on the brain physiology of individuals with ID can be obtained in studies with actigraphy evaluation.

## 6 Conclusion

Our research contributes to the analysis of variations in athletic performance indicators in individuals with ID across the day, about their sleep-wake balance and circadian rhythm. The findings reveal significant differences in performance tests conducted at various times of the day in individuals with ID, particularly noting higher performance levels in the evening compared to the morning. This underscores the importance of considering the circadian rhythm in the timing and planning of sporting activities. The results could aid in the development of more effective training programs and enhance the efficacy of exercises on performance for this population. Therefore, it is recommended to consider the timing of performance tests to maximize the benefits of physical activity in individuals with ID. These findings offer a broad scope for optimizing physical activities for this special population. Our study found that individuals with ID generally possess an intermediate chronotype, but when analyzed alongside performance parameters, they exhibited characteristics of an eveningness type. This suggests a need for developing a distinct chronotype scale for individuals with ID. Furthermore, very few studies have explored the chronotypes of individuals with mild and severe ID, indicating a significant gap in the literature. A thorough analysis of hypothalamus and SCN activities, in line with the classifications of individuals with ID, is deemed necessary. Based on the results of our current study, the timing of physical activity programs for individuals with ID can be adjusted, thereby maximizing the benefits derived from such exercises. In addition, in future studies, inter-gender analysis can be made by conducting research on individuals with ID of both genders. At the same time, a study between individuals with different ID levels is thought to be important in understanding hypothalamic activity in individuals with ID.

## Data Availability

The original contributions presented in the study are included in the article/Supplementary Material, further inquiries can be directed to the corresponding author.
